# Manners of Doctors

**Published:** 1896-02

**Authors:** 


### Manners of Doctors.

A very gratifying tendency has marked the development of the medical profession in the last generation. The slough of mannerisms, the formal dress, the owl-like solemnity, have been thrown off, and the physician, by his own choice, is being judged more by his actual attainments than by external appearances. Thirty years ago a bald head, a white beard and a long frock-coat were as much a part of a physician's equipment as his diploma. Now, on the other hand, it is no infrequent occurrence for an elderly man of real ability, and modern in his methods of practice, to lose a patient through the fear that he may not be fully abreast of the times. What can be further from the old traditions than a leading surgeon lounging about in an outing shirt and blue belt, or a distinguished physician playing polo? Yet these amusements are simply a relaxation from the tension of professional study. One of the best indications that people are learning to judge their medical advisers by their merits is the fact that the advertising physicians are being driven to the wall, despite the most specious extrinsic evidence of success that the shrewdest business methods can produce.—*Lippincot's Magazine.*

